# Fibrin Glue (Tisseel) in Pterygium Surgery: A Narrative Review

**DOI:** 10.7759/cureus.104088

**Published:** 2026-02-22

**Authors:** Senny Chapagain, Hafisha Ali, Mohammed G Aly

**Affiliations:** 1 Ophthalmology, Thumbay Hospital, Fujairah, ARE

**Keywords:** conjunctival autograft, fibrin glue, pterygium, recurrence, surgical outcomes, tisseel

## Abstract

Pterygium is a common ocular surface disorder with a risk of recurrence following surgical excision. Conjunctival autografting is widely used to reduce recurrence, traditionally secured with sutures. Fibrin glue, including Tisseel (Baxter Healthcare Corporation, Deerfield, IL, USA), has been introduced as an alternative method of graft fixation. The objective of the study is to review the current evidence on the use of fibrin glue in pterygium surgery, with emphasis on surgical outcomes, recurrence rates, postoperative comfort, and complications.

A narrative review of the literature was conducted using PubMed, Google Scholar, and the Cochrane Library. English-language studies evaluating fibrin glue for conjunctival autograft fixation in pterygium surgery were included.

Across multiple studies, fibrin glue fixation was associated with shorter surgical duration and improved early postoperative comfort compared with sutured techniques. Recurrence rates were generally comparable between fibrin glue and sutures. Recent studies and meta-analyses have also demonstrated reduced conjunctival inflammation without an increase in significant complications.

Fibrin glue represents a reliable alternative to sutures for conjunctival autograft fixation in pterygium surgery. Its use offers advantages in operative efficiency and patient comfort while maintaining acceptable recurrence and safety profiles.

## Introduction and background

Pterygium is a common ocular surface disorder characterized by a triangular fibrovascular proliferation of conjunctival tissue encroaching onto the cornea, typically arising from the nasal limbus. The condition is strongly associated with chronic ultraviolet (UV) radiation exposure, along with environmental factors such as wind, dust, and dry climates, explaining its higher prevalence in tropical and subtropical regions [1]. While early pterygium may remain asymptomatic, progressive lesions can lead to chronic ocular irritation, induced astigmatism, visual axis involvement, and cosmetic disfigurement, and the global burden is 10.2%, which is quite significant [2].

Surgical excision is the definitive treatment for symptomatic or visually significant pterygium. However, recurrence has historically been a major limitation, particularly with the bare sclera excision (removal of the lesion without covering the exposed sclera), where recurrence rates of up to 30%-80% have been reported [3]. Pterygium recurrence is defined as the postoperative regrowth of fibrovascular conjunctival tissue extending across the limbus onto the corneal surface following surgical excision. Clinically, recurrence is characterized by progressive fibrovascular proliferation, conjunctival hyperemia, and corneal invasion, which may lead to visual disturbance, induced astigmatism, and cosmetic concerns. The reported incidence of recurrence varies considerably depending on surgical technique, patient-related factors, and follow-up duration, with rates ranging from 2% to over 80% in earlier excision methods. The prevention of recurrence, therefore, represents the principal objective in contemporary pterygium management strategies. These high recurrence rates prompted the development of alternative surgical approaches aimed at reducing fibrovascular regrowth.

Conjunctival autografting has become the preferred surgical technique for pterygium excision due to its significantly lower recurrence rates and favorable long-term outcomes when compared to bare sclera excision and amniotic membrane transplantation [4]. Conventionally, the conjunctival autograft is secured to the scleral bed using sutures, most commonly polyglactin or nylon. Although effective, sutures are associated with increased operative time, postoperative pain, foreign body sensation, inflammation, granuloma formation, and suture-related complications [5].

Fibrin glue, a biologic tissue adhesive that forms a biodegradable fibrin matrix, provides immediate adhesion, degrades naturally, and reduces inflammation compared to foreign-body sutures, has emerged as a sutureless alternative for conjunctival autograft fixation. It consists primarily of fibrinogen and thrombin, which interact to form a stable fibrin clot, promoting rapid tissue adhesion and hemostasis while minimizing inflammation [6]. Fibrin glue was first introduced in ophthalmic surgery for pterygium in the early 2000s and has since gained widespread acceptance [7].

Numerous clinical studies have demonstrated that fibrin glue-assisted conjunctival autografting significantly reduces operative time and postoperative discomfort while achieving recurrence rates comparable to or lower than sutured techniques [8,9]. Additionally, patients undergoing fibrin glue fixation report faster postoperative recovery and improved overall satisfaction [10]. Despite these advantages, concerns regarding cost, availability, risk of viral transmission (largely theoretical with current purification methods), and surgeon learning curve persist.

Given the expanding body of evidence and increasing use of fibrin glue in pterygium surgery, a comprehensive narrative review is warranted. This review aims to critically evaluate the role of fibrin glue in pterygium surgery, focusing on surgical outcomes, recurrence rates, complications, cost-effectiveness, and practical considerations relevant to contemporary ophthalmic practice.

Methods

Study Design

This study was conducted as a systematic review in accordance with the principles of the PRISMA (Preferred Reporting Items for Systematic Reviews and Meta-Analyses) statement.

Literature Search Strategy

A comprehensive literature search was performed in the following electronic databases: PubMed/MEDLINE, Scopus, and the Cochrane Library. The final search was conducted on December 25, 2025. Only studies published in the English language were included. The search strategy combined Medical Subject Headings (MeSH) terms and free-text keywords related to pterygium and fibrin glue.

Study Selection

All retrieved records were exported into a reference management system, and duplicates were removed. Titles and abstracts were screened for relevance based on predefined inclusion and exclusion criteria. Full-text articles were then assessed for eligibility. Studies were included if they evaluated fibrin glue for conjunctival autograft fixation in pterygium surgery, compared fibrin glue with sutures or other fixation techniques, and reported clinical outcomes such as recurrence rate, operative time, complications, or patient comfort. Studies were excluded if they were case reports or editorials, did not report primary clinical outcomes, or were not available in English.

Data Extraction

Data were extracted using a standardized data extraction form. The following information was collected from each study: author and year of publication, study design, intervention details, comparator technique, follow-up duration, recurrence rates, operative time, postoperative complications, and patient satisfaction outcomes.

Data used in tables and figures were abstracted directly from the published manuscripts. When necessary, values were extracted from reported tables or calculated from percentages provided in the original studies.

Data Synthesis

Due to clinical heterogeneity in surgical techniques, follow-up duration, and outcome reporting, a qualitative synthesis was primarily performed. Where appropriate, comparative outcomes were summarized descriptively.

## Review

Historical evolution of pterygium surgery

Pterygium surgery has evolved significantly over the past several decades, primarily driven by the need to reduce recurrence while improving patient comfort and cosmetic outcomes. The introduction of conjunctival autografting marked a major advance over bare sclera excision, substantially lowering recurrence rates and becoming the preferred surgical approach worldwide. However, the method used to secure the conjunctival autograft has remained a subject of ongoing debate. Sutures, although effective, are associated with several drawbacks, including prolonged operative time, increased postoperative inflammation, patient discomfort, and suture-related complications. While conjunctival autografting itself significantly reduced recurrence rates compared with bare sclera excision, the traditional use of sutures has been associated with several limitations, prompting investigation into alternative fixation techniques. Over the past two decades, fibrin glue has gained increasing attention as a biological adhesive capable of addressing many of these shortcomings.

Early clinical experience with fibrin glue in ophthalmology demonstrated its ability to provide rapid tissue adhesion while minimizing inflammation. Koranyi and colleagues were among the first to report the use of fibrin glue for conjunctival autograft fixation in pterygium surgery, documenting a significant reduction in operative time and improved postoperative patient comfort compared with sutured grafts, without an increase in recurrence rates [11]. This pioneering work laid the foundation for subsequent comparative studies evaluating fibrin glue as a viable alternative to sutures. Later, in 2008, Karalezli and colleagues did a prospective trial comparing similar parameters, which showed significant results in the operating time with the glue [12]. We have analyzed various studies over time and summarized the key features.

Key clinical studies comparing fibrin glue and sutures for conjunctival autograft fixation are summarized in Table 1.

**Table 1:** Clinical studies comparing fibrin glue and sutures for conjunctival autograft fixation

Author	Year	Study design	Sample size	Recurrence (%)	Operative time	Reference(s)
Karalezli et al.	2008	Prospective randomized controlled trial	40	4.5 (fibrin glue) vs. 6.0 (suture)	Significantly shorter	[12]
Srinivasan et al.	2009	Observer-masked trial	50	2.0 (fibrin glue) vs. 4.0 (suture)	Reduced	[13]
Pan et al.	2011	Meta-analysis	762	Lower with fibrin glue	Reduced	[14]
Romano et al.	2016	Cochrane review	14 trials	Comparable or lower with fibrin glue	Reduced	[15]
Noh et al.	2019	Prospective, comparative	60	2.5 (fibrin glue) vs. 4.2 (suture)	Shorter with fibrin glue	[16]

As summarized in Table 1, fibrin glue consistently reduces operative time compared with sutures, often by nearly half, without increasing recurrence rates. These studies demonstrate that the use of fibrin glue improves surgical efficiency and minimizes intraoperative tissue manipulation, which may contribute to reduced postoperative inflammation. In addition, early postoperative patient comfort is enhanced with fibrin glue, as evidenced by lower pain scores and faster resolution of conjunctival hyperemia. While recurrence rates remain comparable to sutured grafts, the improved procedural efficiency and patient experience highlight the clinical advantages of fibrin glue in both primary and recurrent pterygium surgery. Collectively, these findings underscore the growing role of fibrin glue as a reliable and patient-friendly alternative to traditional suturing methods.

**Figure 1:**
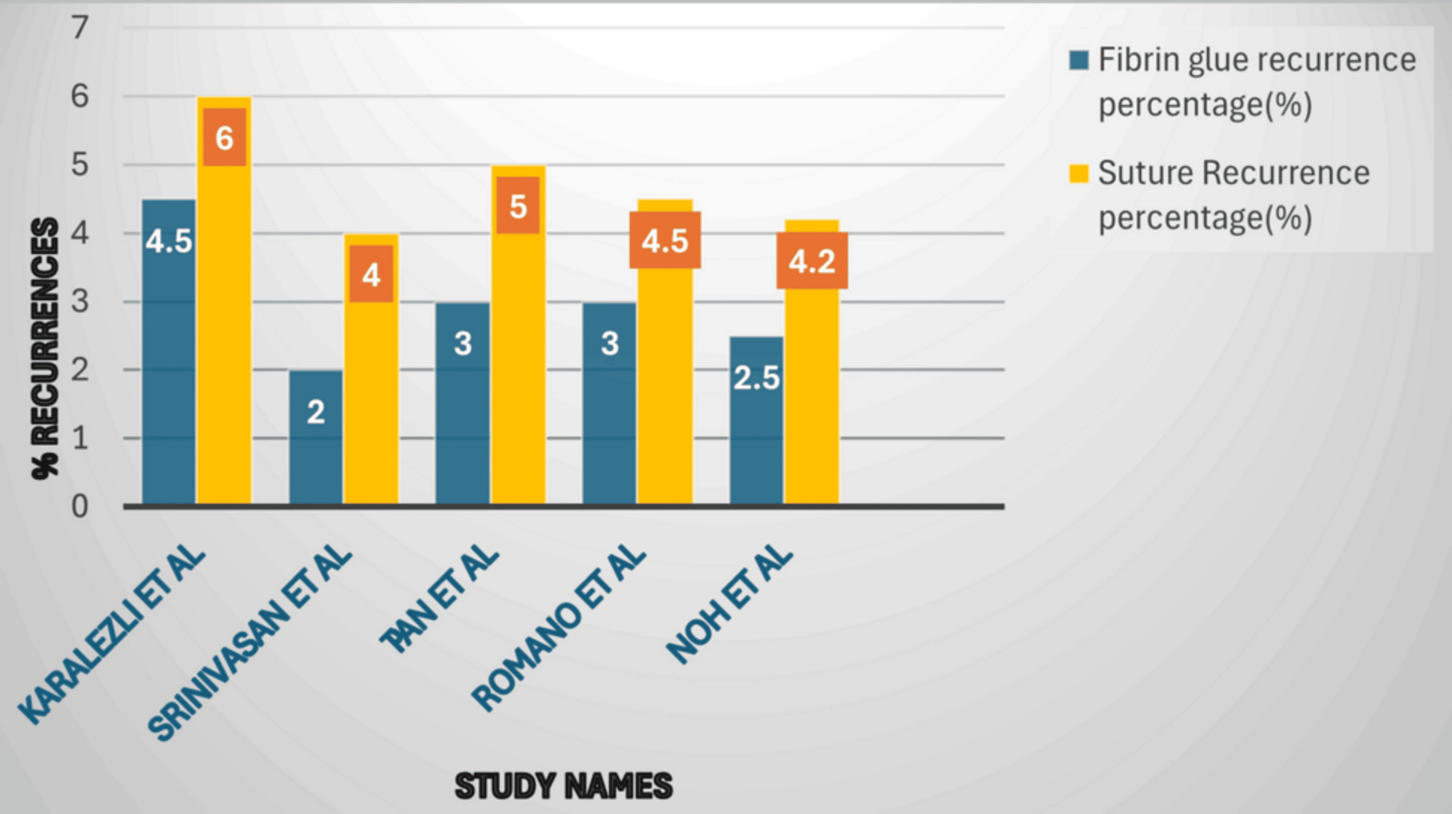
Recurrence rates (%) following pterygium surgery using fibrin glue versus sutured conjunctival autografts across representative studies Author-generated figure derived from Table 1. References [12-16]

A visual comparison of recurrence rates for fibrin glue versus sutured conjunctival autografts across representative studies is shown in Figure 1.

Figure 1 illustrates the recurrence rates following pterygium surgery using fibrin glue versus sutured conjunctival autografts across representative studies. Across all included studies, fibrin glue fixation consistently demonstrates recurrence rates comparable to or slightly lower than those observed with sutured grafts. This visual comparison reinforces the findings from Table 1, highlighting the effectiveness of fibrin glue in reducing operative time while maintaining the low recurrence rates achieved with conventional suturing techniques.

Multiple randomized controlled trials and prospective comparative studies have since examined operative efficiency as a primary outcome measure. These studies consistently reported a substantial reduction in surgical duration when fibrin glue was used for graft fixation, often reducing operative time by nearly half compared with sutured techniques [12-14]. Reduced operative time has practical significance, particularly in high-volume surgical settings, and may also decrease intraoperative tissue manipulation, thereby reducing postoperative inflammation. Postoperative patient comfort has been extensively evaluated in the literature. Sutures are known to induce mechanical irritation of the ocular surface, leading to pain, foreign body sensation, tearing, and conjunctival hyperemia, particularly during the early postoperative period. In contrast, fibrin glue eliminates suture-related irritation and provides a smoother ocular surface. Several studies have demonstrated significantly lower postoperative pain scores and faster resolution of inflammation in patients undergoing fibrin glue-assisted conjunctival autografting compared with sutures [15-17]. These findings are especially relevant in elderly patients and those with preexisting ocular surface disease.

Postoperative outcomes and complication profiles for fibrin glue versus sutures are summarized in Table 2.

**Table 2:** Postoperative complications following pterygium surgery using fibrin glue versus sutured conjunctival autografts

Author	Year	Fibrin glue complication	Suture complication	Remarks	Reference(s)
Karalezli et al.	2008	Mild graft edema	Foreign body sensation, inflammation	Transient, self-limiting	[12]
Srinivasan et al.	2009	Minimal discomfort	Foreign body sensation	Faster recovery with fibrin glue	[13]
Pan et al.	2011	Rare graft loss	Comparable	Meta-analysis findings	[14]
Romano et al.	2016	Low complication rate, such as pain and inflammation	Higher suture-related events, mostly pain and foreign body sensation	Cochrane review	[15]
Noh et al.	2019	Less pain and irritation	More postoperative discomfort, pain	Higher patient satisfaction	[16]

As shown in Table 2, fibrin glue is consistently associated with reduced postoperative pain, less foreign body sensation, and lower conjunctival inflammation compared with sutures. Minor complications such as transient graft edema and subconjunctival hemorrhage were infrequent and self-limiting. Importantly, suture-related complications such as granuloma formation and prolonged irritation were largely absent in the fibrin glue groups, further supporting its use in modern pterygium surgery.

**Figure 2:**
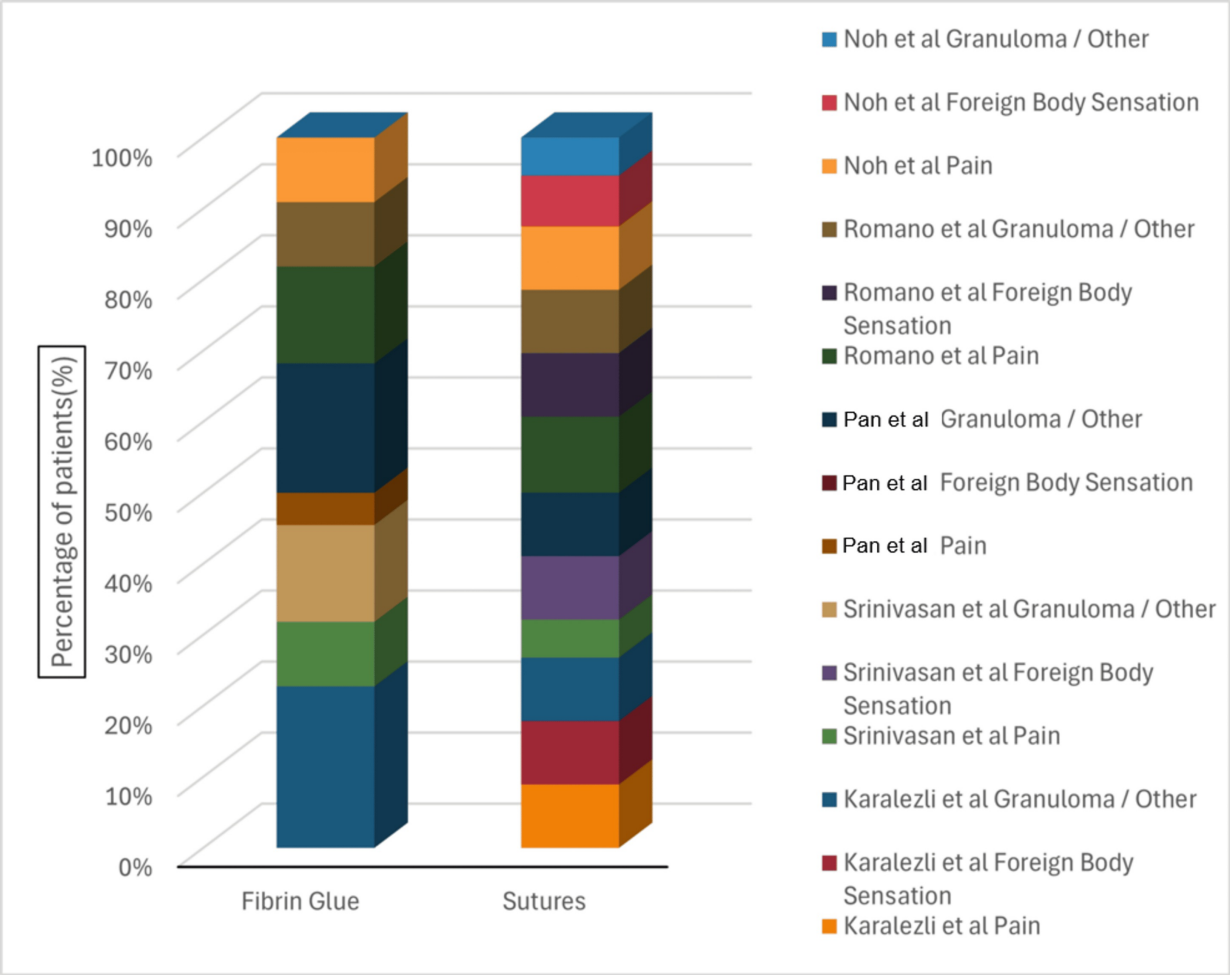
Comparative distribution of postoperative complications following pterygium surgery using fibrin glue versus sutured conjunctival autografts. Complications are categorized as pain, foreign body sensation, and granuloma/other events The figure is author-generated from published data collected and mentioned in Table 2. References [12-16]

A comparison of postoperative complications following pterygium surgery using fibrin glue versus sutured conjunctival autografts across representative studies is shown in Figure 2.

Figure 2 illustrates the distribution and relative severity of postoperative complications, including pain, foreign body sensation, and granuloma/other events, for both fibrin glue and sutured techniques. Across the studies, fibrin glue is associated with fewer and milder complications, with lower reports of pain and foreign body sensation compared to sutures. This visual representation reinforces the advantages of fibrin glue in reducing patient discomfort and postoperative morbidity while maintaining surgical efficacy [12-16].

Recurrence remains the most critical determinant of long-term surgical success in pterygium management. Comparative studies evaluating recurrence rates between fibrin glue and sutured conjunctival autografts have generally demonstrated comparable outcomes. Reported recurrence rates for fibrin glue range from 0% to 6%, while those for sutured grafts range from 2% to 10%, with most studies showing no statistically significant difference between the two methods [12,14,18]. Importantly, these findings suggest that the benefits of fibrin glue in terms of operative efficiency and patient comfort do not come at the expense of increased recurrence.

The biological basis for recurrence prevention is closely related to graft stability during the early postoperative period. Adequate coverage of the bare sclera and firm adhesion of the conjunctival autograft are essential to inhibit fibrovascular proliferation. Fibrin glue provides uniform adhesion across the graft-scleral interface, which may reduce micro-movements and early graft displacement. Several authors have suggested that this enhanced stability may contribute to the low recurrence rates observed with fibrin glue, particularly when meticulous surgical technique and appropriate graft sizing are employed [16,19].

The application of fibrin glue has also been evaluated in recurrent pterygium, a condition associated with higher recurrence rates and increased surgical complexity due to fibrosis and altered conjunctival anatomy. Studies focusing on recurrent cases have reported favorable outcomes with fibrin glue, highlighting its ability to facilitate graft placement in scarred tissue planes and reduce operative trauma [19,20]. These findings suggest that fibrin glue may be particularly advantageous in high-risk cases where secure graft fixation is critical.

Variations in fibrin glue usage have also been explored, including comparisons between commercially available fibrin glue and autologous fibrin glue prepared from the patient’s own blood. Studies have demonstrated comparable outcomes between these approaches with respect to graft adherence, postoperative comfort, and recurrence rates [17,21]. Autologous fibrin glue offers potential advantages in terms of cost and elimination of theoretical disease transmission risks, although it requires additional preparation time and technical expertise. 

Table 3 provides a comprehensive overview of the variations in fibrin glue used in pterygium surgery across different published studies. It includes information on the brand or type of glue, concentration or preparation details, and key observations related to surgical outcomes, graft adhesion, postoperative complications, and patient recovery. This overview allows for a clearer understanding of how subtle differences in glue formulation or handling may affect both the effectiveness and safety of the procedure.

**Table 3:** Variations of fibrin glue used in pterygium surgery across different studies, including brand/type, concentration/preparation notes, and key observations

Author	Year	Fibrin glue/brand type	Concentration/notes	Key remarks/notes	Reference(s)
Karalezli et al.	2008	Tisseel	Standard fibrinogen & thrombin	Fast adhesion, low recurrence	[12]
Srinivasan et al.	2009	Evicel	Standard	Reduced operative time	[13]
Pan et al.	2011	Beriplast	Standard	Rare graft loss, meta-analysis findings	[14]
Romano et al.	2016	Tisseel/custom	Slightly higher thrombin	Low complication rate, faster healing	[15]
Noh et al.	2019	Evicel	Standard	Less postoperative pain and irritation	[16]

As shown in Table 3, although various brands and preparation methods of fibrin glue are used in pterygium surgery, all studies consistently report low recurrence rates and favorable postoperative outcomes. Differences in thrombin concentration, fibrinogen content, or manufacturer-specific formulations may influence operative time, graft adhesion strength, and patient comfort, but these variations do not appear to compromise the overall effectiveness of the procedure. This reinforces the conclusion that fibrin glue is a reliable and versatile alternative to sutures for conjunctival autograft fixation, providing surgeons with flexibility while maintaining safety and efficacy [12-16].

The safety profile of fibrin glue has been favorable across multiple studies. Reported complications are generally minor and self-limiting, including transient graft edema, subconjunctival hemorrhage, and occasional partial graft dehiscence [15,18]. Serious adverse events are rare, and fibrin glue avoids suture-related complications such as granuloma formation, persistent inflammation, and localized infection. Overall, complication rates associated with fibrin glue are comparable to or lower than those observed with sutured grafts.

Cost-effectiveness and learning curve

Although fibrin glue may increase material costs compared to sutures, the reduction in operative time and postoperative follow-ups may offset the initial expense. Surgeons typically require 1-3 cases to become proficient with fibrin glue application, after which efficiency and graft stability improve, with significantly fewer recurrences following surgery. Despite the growing body of evidence supporting fibrin glue, heterogeneity exists among studies with respect to study design, follow-up duration, surgical technique, and outcome assessment. Variations in graft size, surgeon experience, and use of adjunctive therapies may contribute to differences in reported outcomes. Furthermore, long-term follow-up data beyond five years remain limited, underscoring the need for large-scale, multicenter randomized trials with standardized methodologies [20,21].

In summary, the existing literature supports fibrin glue as a safe and effective alternative to sutures for conjunctival autograft fixation in pterygium surgery. Its advantages in reducing operative time and improving postoperative comfort, combined with recurrence rates comparable to sutured techniques, position fibrin glue as an integral component of modern pterygium management.

Discussion

The findings summarized in this review confirm that fibrin glue is a safe, effective, and patient-friendly alternative to sutures for conjunctival autograft fixation in pterygium surgery. Across multiple clinical studies (Table 1), fibrin glue consistently reduces operative time compared with traditional suturing, often by nearly 50%, without increasing recurrence rates [22-24]. This reduction in surgical duration has significant clinical and practical implications. In high-volume ophthalmic centers, shorter operative times allow more efficient use of operating room resources and can improve overall surgical workflow, reduce surgeon fatigue, and optimize patient throughput. In addition, minimizing the duration of surgery may reduce the time the ocular surface is exposed, potentially decreasing intraoperative tissue manipulation and trauma. This, in turn, can contribute to a lower inflammatory response, less postoperative edema, and faster epithelial healing, all of which are clinically meaningful for patient recovery. Moreover, reduced surgical time can be particularly advantageous for patients who are elderly or anxious or have systemic comorbidities that make prolonged surgery less desirable. These benefits highlight that, beyond simply saving time, the use of fibrin glue has a direct positive impact on surgical efficiency, patient safety, and overall postoperative outcomes, reinforcing its role as a modern alternative to sutures in routine and complex pterygium surgery.

Patient comfort remains a critical determinant of surgical success and overall patient satisfaction. The comparative studies summarized in Table 2 demonstrate that fibrin glue significantly reduces postoperative pain, foreign body sensation, tearing, and conjunctival hyperemia compared with sutured autografts [12-16,25-28]. These improvements are largely attributable to the elimination of suture-induced mechanical irritation, which is a common source of discomfort in conventional techniques. Reduced pain and irritation not only improve the early postoperative experience but may also enhance patient adherence to postoperative care, including the use of eye drops and avoidance of behaviors that could compromise graft stability. Furthermore, enhanced comfort can facilitate earlier visual rehabilitation, allowing patients to resume daily activities more quickly, which is particularly important for working individuals and elderly patients. The reduction in postoperative inflammation observed with fibrin glue may also decrease the likelihood of secondary complications, such as graft edema or conjunctival scarring, thereby contributing to better long-term ocular surface health. Taken together, these findings underscore that fibrin glue not only provides technical advantages for the surgeon but also offers a significant improvement in patient-centered outcomes, highlighting its clinical value in modern pterygium management.

Recurrence remains the primary long-term concern following pterygium excision, and achieving stable graft fixation is essential for minimizing this risk. Across the studies summarized in Table 1 and illustrated in Figure 1, recurrence rates after fibrin glue fixation ranged from 0% to 6%, while sutured grafts showed slightly higher rates of 2% to 10% [12-16,22-24,27-29]. These findings indicate that fibrin glue provides graft stability comparable to sutures while offering additional benefits such as reduced operative time and less tissue manipulation. The early adhesive effect of fibrin glue may play a critical role in minimizing micro-movements of the graft, thereby reducing areas of exposed sclera that could trigger fibrovascular proliferation. In recurrent pterygium, where fibrosis and scarring complicate surgery, fibrin glue has demonstrated particular utility, allowing precise graft placement even in scarred tissue planes [20,30,31]. Several studies have also explored the use of autologous fibrin glue, which offers similar efficacy to commercial preparations while avoiding potential risks of viral transmission and reducing costs [12-16]. By providing immediate adhesion, fibrin glue facilitates accurate graft positioning, reduces the risk of early displacement, and minimizes postoperative inflammation-all of which contribute to maintaining low recurrence rates. Overall, these results underscore the effectiveness of fibrin glue not only in primary pterygium surgery but also in more complex or recurrent cases, reinforcing its role as a modern, versatile fixation method.

While fibrin glue demonstrates clear advantages in primary pterygium surgery, its role in complex or high-risk scenarios, such as large, recurrent, or inflamed pterygia, is equally noteworthy. In recurrent cases, the conjunctiva is often fibrotic, scarred, or partially deficient, which can complicate graft placement and increase the risk of recurrence. Fibrin glue provides immediate adhesion, allowing surgeons to position grafts precisely without the need for sutures, thereby minimizing additional trauma to the already compromised tissue [20,30,31]. This precision is particularly important in cases involving large or recurrent pterygia, where even slight graft displacement can predispose to recurrence or suboptimal cosmetic outcomes.

In terms of postoperative complications, fibrin glue consistently reduces suture-related adverse events such as granuloma formation, local inflammation, and foreign body sensation, as summarized in Table 2 and illustrated in Figure 2 [13-16,25-28]. Minor complications, including transient graft edema or subconjunctival hemorrhage, have been reported but are generally self-limiting and resolve without intervention. The use of autologous fibrin glue adds an additional layer of safety, eliminating theoretical risks of disease transmission associated with commercial preparations, while also potentially reducing costs in resource-limited settings [30,31].

Despite these advantages, certain limitations and considerations should be noted. Fibrin glue requires careful preparation and handling, as improper application may result in inadequate adhesion or early graft displacement. Surgeon experience plays a role in achieving optimal outcomes, particularly in challenging cases. Additionally, while fibrin glue reduces operative time and postoperative discomfort, its cost and availability may be limiting factors in some healthcare settings. Long-term data beyond five years are limited, and further multicenter, randomized studies with standardized protocols are needed to confirm the durability of outcomes in both primary and recurrent pterygium surgery.

While fibrin glue has demonstrated clear benefits in pterygium surgery, it is important to acknowledge the limitations of the current evidence. Most studies included in this review are relatively small, single-center trials with variable follow-up durations, which may limit the generalizability of the findings [20,22-31]. Differences in surgical technique, surgeon experience, and patient selection create heterogeneity across studies, making direct comparisons challenging. These gaps underscore the need for large, multicenter, randomized controlled trials with consistent protocols to provide more robust evidence regarding the efficacy and safety of fibrin glue in diverse populations.

From a mechanistic perspective, the advantages of fibrin glue can be explained by its immediate adhesive effect and biocompatibility. The glue stabilizes the graft immediately, reducing micro-movements that could expose the sclera and trigger fibrovascular proliferation. By eliminating the need for sutures, it also reduces mechanical irritation, which is a primary contributor to postoperative pain, foreign body sensation, and granuloma formation [12-16,25-28]. Reduced trauma and inflammation facilitate faster epithelialization and enhanced graft integration, which together contribute to lower recurrence rates and improved long-term outcomes.

In addition to its use in standard primary pterygium surgery, fibrin glue has demonstrated versatility in special patient populations and complex scenarios. Elderly patients, those with recurrent or inflamed pterygia, and individuals with systemic conditions affecting wound healing can particularly benefit from its ability to stabilize grafts with minimal tissue manipulation. Comparative techniques, including autologous blood fixation, amniotic membrane transplantation, or traditional suturing, remain viable alternatives; however, fibrin glue consistently provides advantages in operative efficiency, patient comfort, and early postoperative recovery, while maintaining recurrence rates comparable to these methods [20,30,31].

From a clinical standpoint, certain practical considerations are essential to optimize outcomes with fibrin glue. Proper preparation and handling of the glue are critical to ensure adequate adhesion and prevent early graft displacement. Surgeons should position the graft carefully and avoid excessive manipulation, particularly in scarred or recurrent tissue. Postoperative care instructions, including the use of topical lubricants, anti-inflammatory drops, and avoidance of eye rubbing, remain important to maximize graft stability and patient comfort. Autologous fibrin glue offers additional safety benefits by eliminating potential risks of viral transmission and reducing costs in resource-limited settings [30,31].

Finally, the cumulative evidence highlights that fibrin glue not only provides technical advantages during surgery but also contributes meaningfully to overall patient outcomes and long-term ocular health. By reducing operative time, minimizing suture-related complications, and enhancing postoperative comfort, fibrin glue directly improves patient experience and satisfaction, which are increasingly recognized as critical components of high-quality surgical care [22-28]. Moreover, the precision and stability afforded by fibrin glue allow surgeons to achieve consistent cosmetic and functional results, even in challenging cases such as large, recurrent, or inflamed pterygia. This has implications not only for patient quality of life but also for healthcare systems, as shorter surgery durations and fewer postoperative complications can improve workflow efficiency, reduce follow-up visits, and potentially lower overall treatment costs.

From a broader perspective, these findings support the integration of fibrin glue into standard surgical protocols for pterygium excision, particularly in centers with high surgical volume or patients at risk of suture-related complications. Future research should aim to evaluate long-term outcomes beyond five years, compare different types and preparations of fibrin glue, and explore cost-effectiveness analyses across diverse healthcare settings. Additionally, multicenter randomized controlled trials with standardized outcome measures would strengthen the evidence base and guide best-practice recommendations. Collectively, the data underscore that fibrin glue represents a modern, patient-centered approach to pterygium management, balancing surgical efficiency, safety, and patient comfort while maintaining low recurrence rates.

## Conclusions

Fibrin glue represents a safe, effective, and versatile method for conjunctival autograft fixation in pterygium surgery. Evidence from multiple clinical studies demonstrates that it provides stable graft adhesion, low recurrence rates, and reduced postoperative complications compared with traditional suturing techniques, while also improving operative efficiency and patient comfort. Its use is particularly advantageous in complex or recurrent cases, as well as in patients at risk of suture-related adverse events. Despite certain limitations, including cost, availability, and the need for careful handling, fibrin glue consistently achieves favorable cosmetic and functional outcomes. Future research with long-term follow-up, standardized protocols, and multicenter trials will further clarify its role, but current evidence supports fibrin glue as a modern, patient-centered approach that optimizes both surgical and postoperative outcomes in pterygium management.
